# Downregulation of hypermethylated in cancer-1 by *miR*-*4532* promotes adriamycin resistance in breast cancer cells

**DOI:** 10.1186/s12935-018-0616-x

**Published:** 2018-09-04

**Authors:** Fan Feng, Xiaolan Zhu, Chunyan Wang, Liang Chen, Weiping Cao, Yueqin Liu, Qi Chen, Wenlin Xu

**Affiliations:** 1 The Fourth Affiliated Hospital of Jiangsu University, 20 Zhengdong Road, Zhenjiang, 212001 People’s Republic of China; 2 The Medical College of Jiangsu University, 301# Xuefu Road, Zhenjiang, 212013 People’s Republic of China; 3 The Institute of Life Science, Jiangsu University, 301# Xuefu Road, Zhenjiang, 212013 People’s Republic of China

**Keywords:** *miR*-*4532*, Breast cancer, Multidrug resistance, Hypermethylated in cancer-1, Interleukin-6/signal transducer and activator of transcription 3 pathway

## Abstract

**Background:**

MicroRNAs are small RNAs (~ 22 nt) that modulate the expression of thousands of genes in tumors and play important roles in the formation of multidrug resistance. In this study, we firstly investigated that *miR*-*4532* involved in the multidrug resistance formation of breast cancer by targeting hypermethylated in cancer 1 (*HIC*-*1*), a tumor-suppressor gene.

**Methods:**

To identify and characterize the possible miRNAs in regulating multidrug resistance, we employed the transcriptome sequencing approach to profile the changes in the expression of miRNAs and their target mRNAs were obtained by bioinformatics prediction. Then the molecular biology experiments were conducted to confirm *miR*-*4532* involved in multidrug resistance formation of breast cancer.

**Results:**

The luciferase reporter assay experiment was employed to confirm that *HIC*-*1* was the target of *miR*-*4532*. Transfection with an *miR*-*4532* mimic indicated *miR*-*4532* mimic significantly increased breast cancer cell resistance to adriamycin. Cell proliferation and invasion assay experiments showed overexpression of HIC-1 inhibited the invasion and metastasis of breast cancer cells. Meanwhile, the interleukin (IL)-6/signal transducer and activator of transcription 3 (STAT3) signaling pathway was confirmed to be involving in multidrug resistance by western blotting experiments.

**Conclusions:**

These results suggest that downregulation of hypermethylated in cancer-1 by *miR*-*4532* could promote adriamycin resistance in breast cancer cells, in which the IL-6/STAT3 pathway was regulated by the HIC-1. This finding might contribute to new therapeutic target for reversal of tumor resistance.

**Electronic supplementary material:**

The online version of this article (10.1186/s12935-018-0616-x) contains supplementary material, which is available to authorized users.

## Background

Breast cancer is one of the most common types of malignant tumors in women and is the main cause of cancer-related death in women [1]. Based on GLOBOCAN estimates, there were about 1,671,149 new cases of breast cancer worldwide in 2012, of which 521,907 patients died of breast cancer [2]. According to the statuses of the estrogen receptor (ER), progesterone receptor (PR), and human epidermal growth factor receptor-2 (HER-2), breast cancer can be classified into four distinct molecular subtypes, including luminal-type, basal type, HER-2 positive, and normal [3]. Although significant achievements have been made in chemotherapeutic strategies, the acquisition of resistance to adriamycin or other chemotherapeutic drugs is the major clinical obstacle to successful treatment [4]. Consequently, it is necessary to elucidate the regulatory mechanisms of drug resistance, which will be helpful for designing new and targeted therapeutic strategies that can overcome drug resistance and improve the clinical value of treatments for breast cancer.

MicroRNAs (miRNAs) are highly conserved, endogenous, noncoding RNAs about 22 nucleotides in length. These molecules bind to mRNAs of at the 3′-untranslated region (3′-UTR) with perfect or near-perfect complementarity, resulting in degradation or inhibition of the target gene. The profile of miRNAs was proved to be closely associated with the tumor deterioration and metastasis [5]. And also the expression of miRNAs could be regulated by the environmental factor such as hypoxia in cancer cells [6]. Interestingly, one miRNA can modulate hundreds of target genes, and one gene can be repressed by hundreds of miRNAs [7, 8]. These miRNA-based pathways have been shown to regulate cell proliferation, migration and programmed cell death, including apoptosis and autophagy, in cancer cells [9–12]. In mammals, many studies have shown that miRNAs, such as *miR*-*365*, *miR*-*125b*, *miR*-*491*, and *miR*-*133a*, play important roles in multidrug resistance (MDR) [13–15]. However, few studies have examined the signaling mechanisms through which miRNAs are involved in the regulation of MDR [16]. The roles of miRNAs in apoptosis, autophagy, and necroptosis have been examined, with a focus on the impact of these regulatory activities on cancer progression [17, 18]. Moreover, several miRNAs, including *miR*-*27*, have been shown to be involved in MDR in cancer [19], particularly through modulation of apoptosis and autophagy pathways.

*miR*-*4532* has been shown to be differentially expressed in resistant and sensitive breast cancer cells by next-generation sequencing [20]. Bioinformatics analysis of *miR*-*4532* has shown that hypomethylated in cancer-1 (HIC-1) may be an miRNA target gene involved in the regulation of resistance of cancer cells to chemotherapeutic drugs. The *HIC*-*1* gene, located on chromosome 17p13.3, is a tumor-suppressor gene that is frequently silenced or deleted in a variety of human cancers, such as leukemia, liver cancer, pancreatic cancer, and breast cancer [21–24]. HIC-1 is involved in several complex biological functions in the regulation of drug resistance in cancer, including cell survival, cell growth, cell motility, and cell migration [25]. Many downstream targets of HIC-1 responsible for development, proliferation, and migration, including sirtuin-1, C-X-C chemokine receptor type 7, transcription factor 4, matrix metalloproteinase (MMP) 2, MMP9, and cyclin D1, have been identified [26–30]. However, the mechanisms regulating HIC-1 have not been reported, particularly with regard to how miRNAs regulate HIC-1 in breast cancer cells.

Therefore, in this study, we aimed to elucidate the effects of *miR*-*4532* on the regulation of HIC-1 during acquisition of MDR in breast cancer. Our findings provided important insights into the mechanisms through which *miR*-*4532* regulates HIC-1 expression to affect drug resistance in breast cancer cells.

## Materials and methods

### Cell lines and cell culture

MCF-7 and MDA-MB-231 human breast cancer cells and 293-T cells were maintained in our laboratory. Adriamycin-resistant MCF-7/ADR and MDA-MB-231/ADR cells were established by induction with gradient concentrations of adriamycin in vitro. The induction method is as follows: using a gradient culture of 0.1, 0.2, 0.4, 0.6, 0.8, 1.0 μg/ml adriamycin concentrations, each round screened the surviving cells for the beginning of the next drug resistance concentration, until the cells surviving in 1 μg/ml were MCF-7/ADR and MDA-MB-231/ADR. Cells were cultured in RPMI-1640 (Gibco, USA) supplemented with 10% fetal calf serum (Gibco) and 1% penicillin and streptomycin (Invitrogen, Carlsbad, Ca, USA) at 37 °C in a humidified atmosphere with 5% CO_2_. To maintain the ADR-resistant phenotype, adriamycin was added to the culture medium at a final concentration of 1 μg/ml, and MCF-7/ADR and MDA-MB-231/ADR cells were cultured for 2 weeks in ADR-free medium prior to their use in experiments.

### Human tissue specimens and survival curves

Ten pairs of breast tumor specimens and matched adjacent nontumor tissues were randomly obtained from patients who had undergone mastectomy at the Fourth Affiliated Hospital of Jiangsu University. Informed consent was obtained from all patients, and the study was approved by the Ethics Committee of the Fourth Affiliated Hospital of Jiangsu University and was carried out in strict accordance with the Declaration of Helsinki.

Survival curves were calculated using the Kaplan–Meier method, conducted with the R Bioconductor ‘survival’ package. Kaplan–Meier curves were generated using a database of public microarray datasets (http://kmplot.com) via website interface 2015.

### miRNA extraction, next-generation sequencing, and quantitative real-time reverse transcription polymerase chain reaction (qRT-PCR)

Small RNAs were extracted from MCF-7/ADR and MCF-7 cells using RISO RNA ISOlation Reagent (Biomics, USA) according to the manufacturer’s instructions, and the samples were placed in dry ice for delivery to Genesky Biotechnologies Inc. (China) for next-generation sequencing analysis of miRNAs. The expression levels of mature miRNAs were then analyzed with a stem-loop kit and qRT-PCR, which was conducted using TaqMan Universal PCR Master Mix, as described by kit instruction. U6 was used as an endogenous control for data normalization, and all reactions were run in triplicate. The miRNA was calculated using the 2^−∆∆Ct^ method, where $\Delta \Delta {\text{Ct}}\, = \,\left( {{\text{Ct}}_{\text{miRNA}} \,{-}\,{\text{Ct}}_{\text{internal reference}} } \right)_{\text{experiment}} \,{-}\,\left( {{\text{Ct}}_{\text{miRNA}} \,{-}\,{\text{Ct}}_{\text{internal reference}} } \right)_{\text{control}}$ [31].

### Target gene prediction and gene ontology (GO) analysis of miRNAs

Target gene prediction of differentially expressed miRNAs obtained from sequencing data was performed using miRBase (http://mirbase.org/index.shtml), TargetScan (http://www.targetscan.org/), and Tarbase (http://microrna.gr/tarbase/) databases. According to the annotations of the predicted proteins from UniProt knowledgebase (http://www.expasy.org/sprot/), corresponding GO IDs of these proteins were obtained by InterproScan searching (http://www.ebi.ac.uk/InterProScan/). According to the methods described by Ye et al. [32] and based on the Gene Ontology Database (OBO v1.2format: http://www.geneontology.org/GO.downloads.ontology.shtml), the GO classifications of the proteins were determined using WEGO (http://wego.genomics.org.cn/).

### Cell proliferation assay

Cell Counting Kit 8 (CCK-8) assays were conducted as follows. Briefly, 1000 cells from each group were plated in each well of a 96-well microplate in 150 μl medium with different concentrations of chemotherapeutic drugs. After 48 h of culture, 10 μl of CCK-8 solution was added to the medium, and the cells were incubated for 3 h at 37 °C. The optical density at 570 nm was measured with a microplate spectrophotometer. Three independent experiments were performed, and half-maximal inhibitory concentration (IC_50_) was derived using the curve fitting method.

### Cell invasion assays

The cells were harvested 72 h after transfection with siRNA-HIC-1 or control RNA and were resuspended in medium. The cells were then plated at a density of 2.0 × 10^6^ cells/ml. In total, 0.2 ml cells was added to the upper chamber of transwell chambers (24-well inserts, 8-µm pore size; Millipore, Bedford, MA, USA), and 0.6 ml medium containing 10% fetal bovine serum was added to the lower chamber as a chemoattractant.

### Cell apoptosis detection by FCM (flow cytometry)

Apoptotic cells differentiated from viable or necrotic ones were analyzed by combined application of propidium iodide (PI) and annexin V-APC as described [31]. Samples were washed twice and adjusted at a concentration of 1 × 10^6^ cells/ml with ice-cold PBS. A total of 100 μl suspension was added into each Falcon tube, and 10 μl of PI (20 μg/ml) and 10 μl of annexin V-APC were added into the labeled tubes. Cells were incubated at room temperature in the dark for at least 30 min, then PBS binding buffer about 400 μl was added into each tube without washing, and analyzed by the FACSCalibur™ Flow Cytometer (BD Biosciences) as soon as possible (within 30 min).

### qRT-PCR analysis of mRNA expression

Total mRNA was extracted from breast tumor tissues, matched adjacent nontumor tissues, and cell lines using TRIzol reagent (Invitrogen) according to the manufacturer’s protocol. cDNA was then synthesized using a RevertAid First-Strand cDNA Synthesis Kit (Fermentas, USA). The expression levels of each analyte compared with untreated controls were assessed using the 2^−∆∆Ct^ method. All experiments were conducted at least in triplicate. The primers used to detect mRNA expression are listed in Additional files 1, 2 and 3.

### Small RNA transfection

The *miR*-*4532* mimic (5′-UGUAAACAUCCUACACUCUCAGC-3′) was purchased from a domestic provider in China (Genepharma, Shanghai, China). Cells were plated into 6-well plates at a density of 1 × 10^5^ cells/well. After 24 h, 80 nM *miR*-*4532* mimic and its negative control were transfected into cells using Lipofectamine 2000 reagent (Invitrogen) according to the manufacturer’s protocol. The transfected cells were then harvested for studies after culturing for 48 h. Three independent experiments were performed.

### Luciferase reporter assay

For luciferase reporter experiments, the 3′-UTR sequence of *HIC*-*1* was amplified by PCR from human genomic DNA using primers that included *XbaI* and *EcoRI* sites. Primers for *HIC*-*1* 3′-UTR were as follows: forward, 5′-CTAGTCTAGACTCTGTCGCTGCTGCGCGGCCCTGG-3′ and reverse, 5′-CCGGAATTCTCGCAAGGGCCGGAGGTAGGGCTAG-3′. The PCR products were ligated into the luciferase UTR-report vector (Promega, USA), and mutations within the putative *miR*-*4532* binding sites were introduced using the following primers: luc-HIC1-mut-RP, GGGCCCCTTGTCCCGCGACCCCCGAGCTAAGG and luc-HIC1-mut-FP, CGGGACAAGGGGCCCACGGGGGTGGGATGGGG. Cells were transfected with the UTR-report vector, 20 ng control Renilla luciferase pRL-TK vector (Promega), and 10 nM *miR*-*4532* mimic for the *HIC*-*1*-3′-UTR construct using Lipofectamine 2000 reagent. Forty-eight hours after transfection, cells were lysed with a 1× passive lysis buffer, and assays were performed using a Dual-Luciferase Reporter Assay System kit (Promega) according to the manufacturer’s instructions.

### Western blot analysis

Total cellular extracts were prepared by homogenization of 3 × 10^6^ to 5 × 10^6^ cells in radioimmunoprecipitation assay buffer (Beyotime, China). Western blot analysis was performed as described previously [33]. After separation by sodium dodecyl sulfate polyacrylamide gel electrophoresis on 15% gels, the gels were immersed in cold transfer buffer (0.025 M Tris, 0.19 M glycine, 20% methanol), and the proteins were transferred to polyvinylidene difluoride membranes. The membranes were then blocked in 3% skim milk powder in PBS Tween-20 (PBST) overnight at 4 °C and immunoblotted with primary antibodies (Cell Signaling Technology, USA) diluted 1:1000 for 1.5 h at room temperature. After washing five times with PBST, the membranes were incubated with peroxidase-conjugated goat anti-rabbit IgG diluted 1:2000 for 1.5 h at room temperature. After washing with PBS five times, the bands were visualized using diaminobenzene or enhanced chemiluminescence (ECL; Thermo, Shanghai, China).

### Statistical analysis

Data are shown as means ± standard deviations. Statistical significance between groups was evaluated using Student’s t-tests in SPSS PASW Statistics version 18 Multilingual (SPSS Inc., USA). Results with *p* value of less than 0.05 were considered statistically significant.

## Results

### Global identification of differentially expressed miRNAs in drug-resistant and -sensitive breast cancer cell lines

**Fig. 1:**
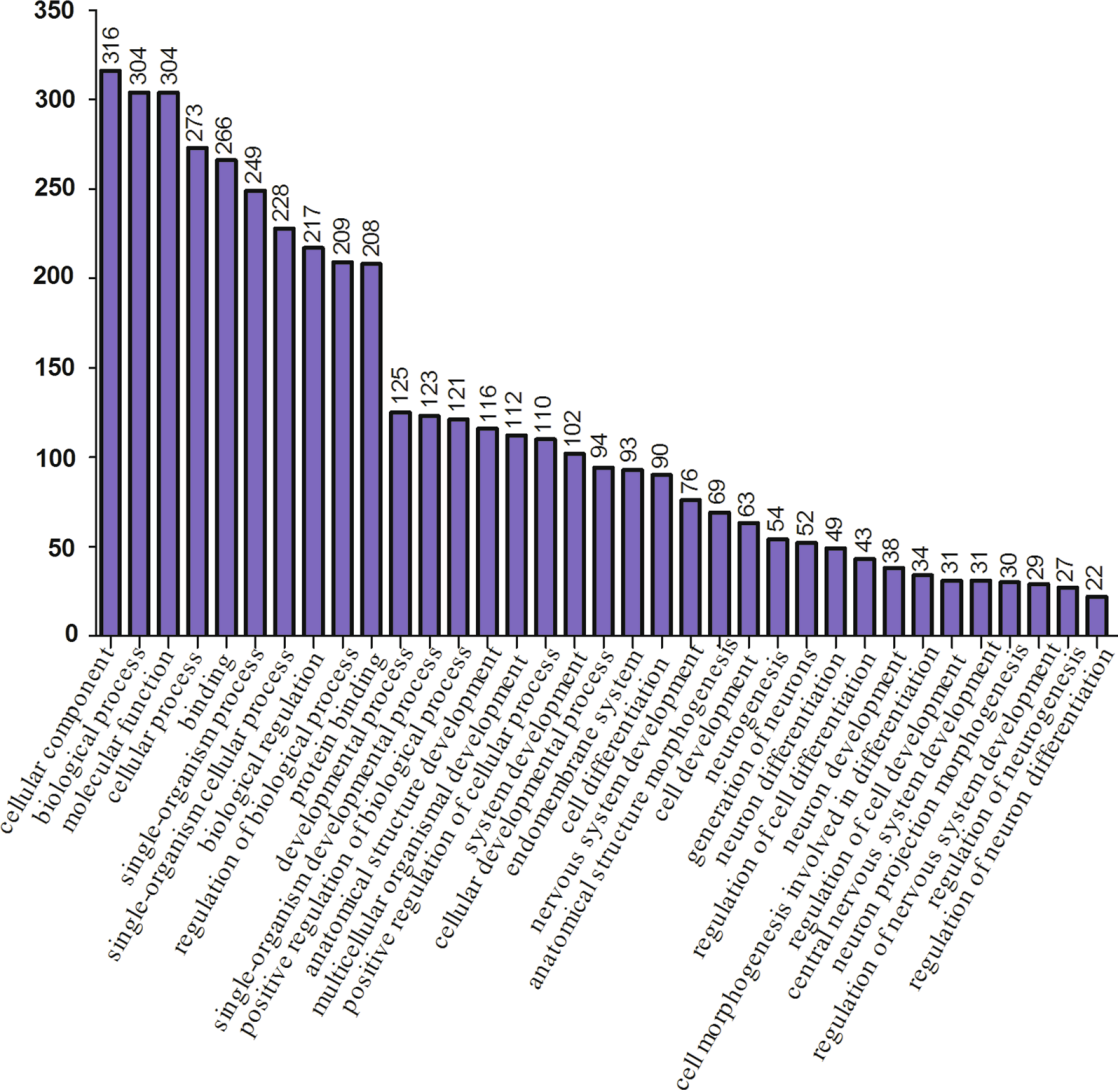
GO analysis of target genes predicted according to differential expression of miRNAs. The X-axis indicates the function of each GO annotation, and the Y-axis represents the relative expression of proteins for every GO annotation

To elucidate the multidrug resistance mechanisms of breast cancer at the miRNA level, we employed next-generation sequencing to globally identify differentially expressed miRNAs. By comparing the miRNA expression profiles of drug-resistant and control cells (MCF-7/ADR and MCF-7, respectively), five miRNAs were screened to determine significant differences in expression (Table 1). Through target gene prediction in miRBase, TargetScan, and Tarbase, a list of target genes was obtained (Additional file 1). GO analysis was performed to determine the physiological roles of these target genes (Fig. 1), and the results indicated that these target genes were mainly involved in regulation of defense response, biological processes, cellular components, neuronal differentiation, molecular functions, and the ephrin receptor signaling pathway.

**Table 1:** The known miRNA of differential expression between MCF-7/ADR and MCF-7 cells

miRNA	log2 fold change	p value	Mature sequences
miR-4532	2.264916005	0.023517834	ccccggggagcccggcg
miR-30c	− 2.520599038	0.011715526	uguaaacauccuacacucucagc
miR-4485	− 2.646290825	0.008137982	uaacggccgcgguacccuaa
miR-6087	− 2.319108533	0.020389152	ugaggcgggggggcgagc
miR-30b	− 2.197801761	0.027963236	uguaaacauccuacacucagcu

### Verification of differentially expressed miRNAs in MDA-MB-231 cells by RT-PCR

**Fig. 2:**
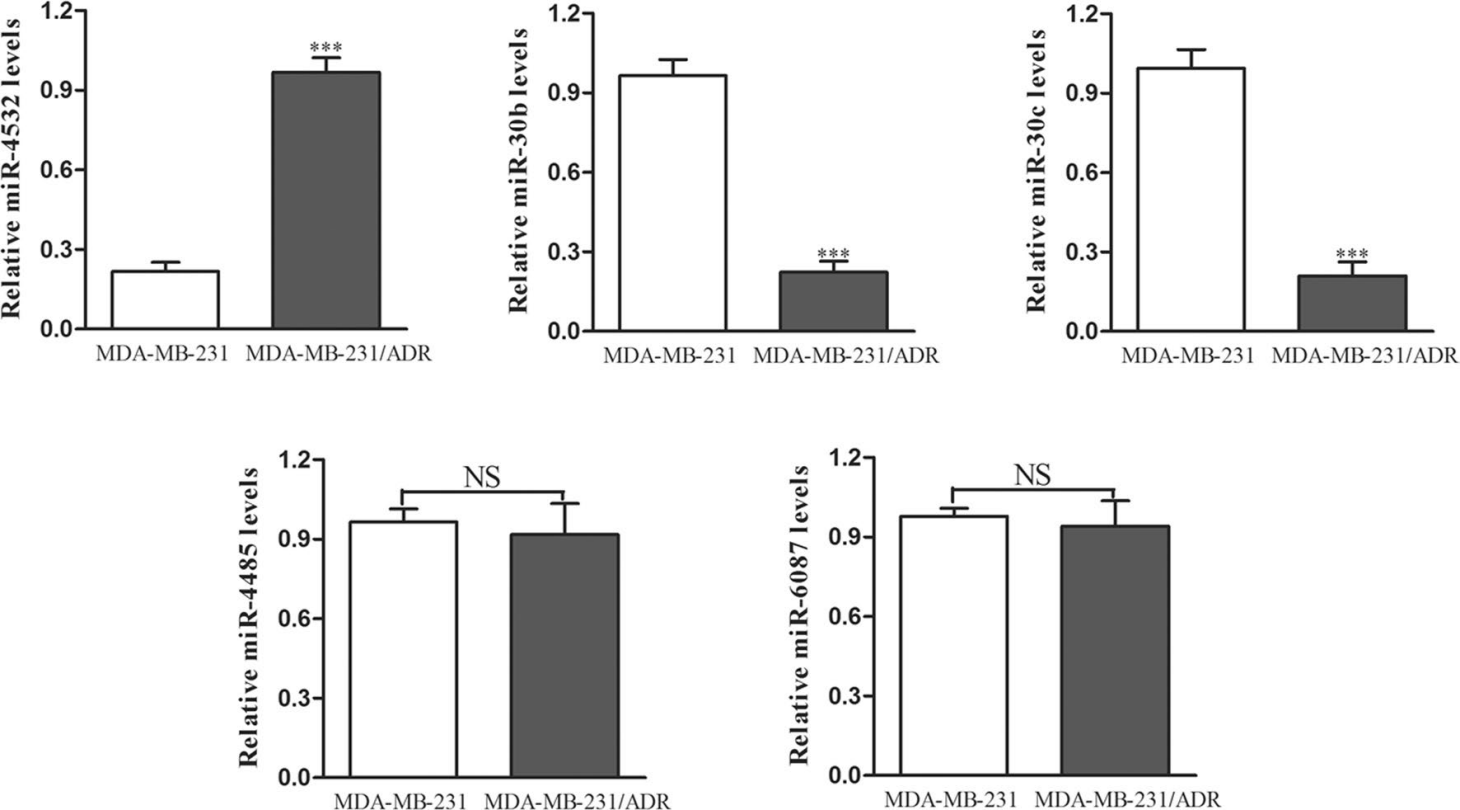
qPCR analysis of differentially expressed miRNAs in MDA-MB-231 cells. *NS* not significant; ***p < 0.01

Next, we validated the differences in miRNA expression in MDA-MB-231 and MDA-MB-231/ADR cells using RT-PCR with the primers listed in Additional file 2. The results showed that *miR*-*4532*, *miR*-*30b*, and *miR*-*30c* expression levels were similar to those in MCF-7 cells. *miR*-*30b* and *miR*-*30c* have been reported in prior studies of cancer drug resistance. Therefore, we focused on *miR*-*4532* as a potential target miRNA regulating drug resistance in breast cancer cells (Fig. 2).

### Overexpression of *miR*-*4532* increased adriamycin resistance in MCF-7 cells

**Fig. 3:**
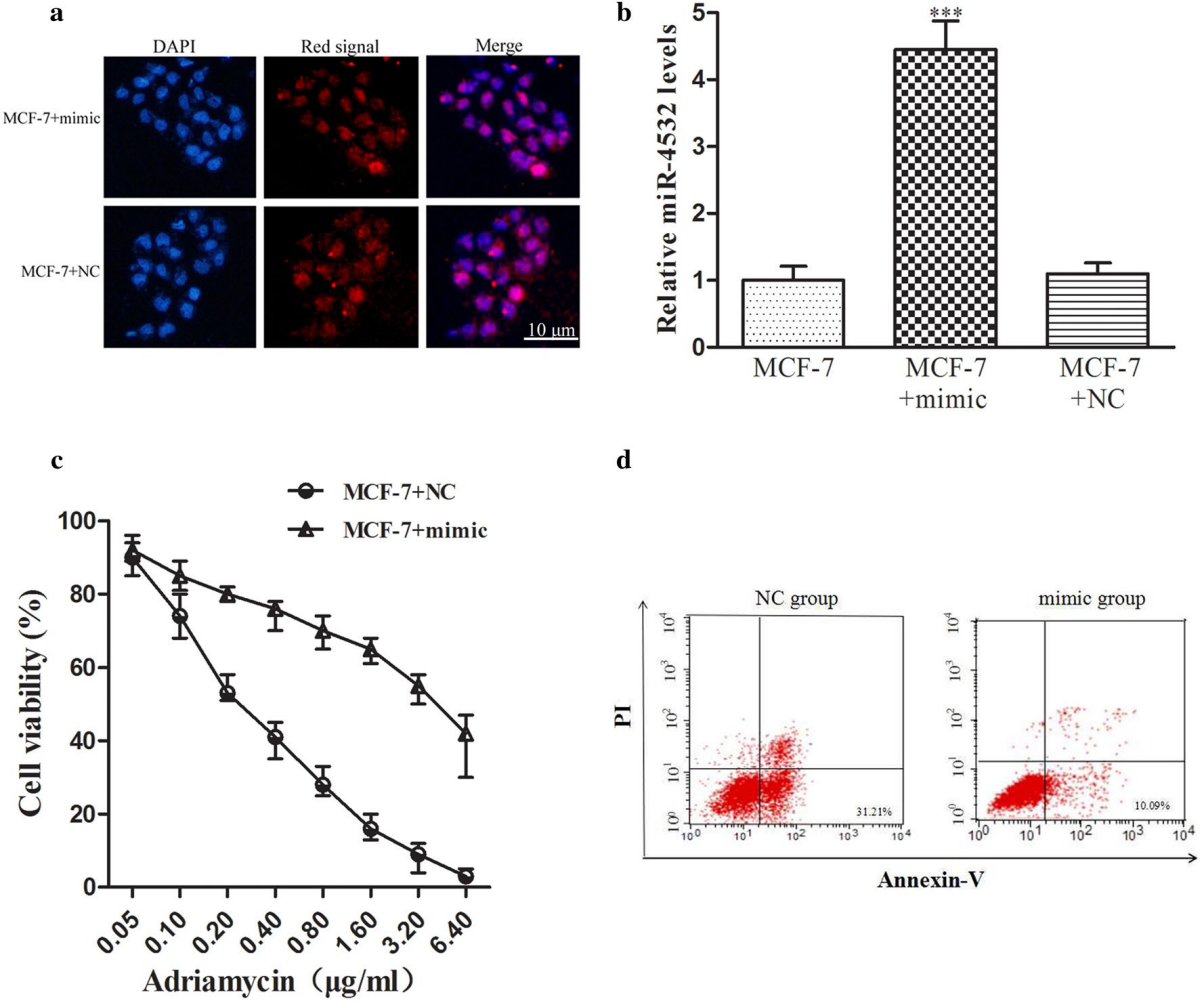
Overexpression of *miR*-*4532* increased adriamycin resistance in MCF-7 cells. **a** After transfection with *miR*-*4532* negative control (red), the cells were washed three times with PBS and then observed under a fluorescence microscope with a ×400 lens. **b** qPCR results showed that the expression of *miR*-*4532* was significantly increased in MCF-7 cells transfected with the *miR*-*4532* mimic (***p < 0.01, Student’s *t*-tests). **c** After transfection with *miR*-*4532* mimic or negative control (NC) for 48 h, MCF-7 cells were subsequently treated with various concentrations of adriamycin for 48 h. Cell viability was determined using CCK-8 assays, and the calculation of IC_50_ was obtained by using Probit regression analysis in SPSS 18.0 software (IC_50_ for mimic and negative control group was 3.457 ± 0.274 and 0.603 ± 0.108 μg/ml, respectively, *p* < 0.05). **d** Overexpression of *miR*-*4532* in MCF-7 cells rescued adriamycin-induced apoptosis after 48 h of treatment with 1 μM adriamycin, the rate of early apoptosis of NC group and mimic group was 31.21% and 10.09%, respectively, *p* < 0.01

To investigate whether *miR*-*4532* modulated chemosensitivity in breast cancer, MCF-7 cells were transfected with 60 nM *miR*-*4532* mimic or negative control, and there was no significant difference in the transfection efficiency between the control group and the experimental group (Fig. 3a). Real-time PCR revealed *miR*-*4532* was efficiently transfected into cells (Fig. 3b). CCK-8 assays showed that the drug resistance index of MCF-7 cells transfected with the *miR*-*4532* mimic was about 5.5 times higher that of cells transfected with the control miRNA mimic (IC_50_: 3.457 ± 0.274 and 0.603 ± 0.108 μg/ml, respectively, the calculation of IC_50_ is obtained by using Probit regression analysis in SPSS 18.0 software; *p* < 0.05; Fig. 3c), suggesting that *miR*-*4532* could mediate resistance to adriamycin in breast cancer cells. Furthermore, flow cytometry analysis indicated that *miR*-*4532* transfection decreased apoptosis in MCF-7 cells compared with that in the negative control in response to adriamycin treatment, the rate of early apoptosis of NC group and mimic group was 29.69% ± 0.78% and 10.81% ± 2.13%, respectively (Additional file 3), as shown in Fig. 3d (31.21% vs 10.09%, *p* < 0.01). These results demonstrated that *miR*-*4532* restoration could obviously increase the resistance of MCF-7 cells to adriamycin.

### Target gene prediction and experimental verification of *miRNA*-*4532*

**Fig. 4:**
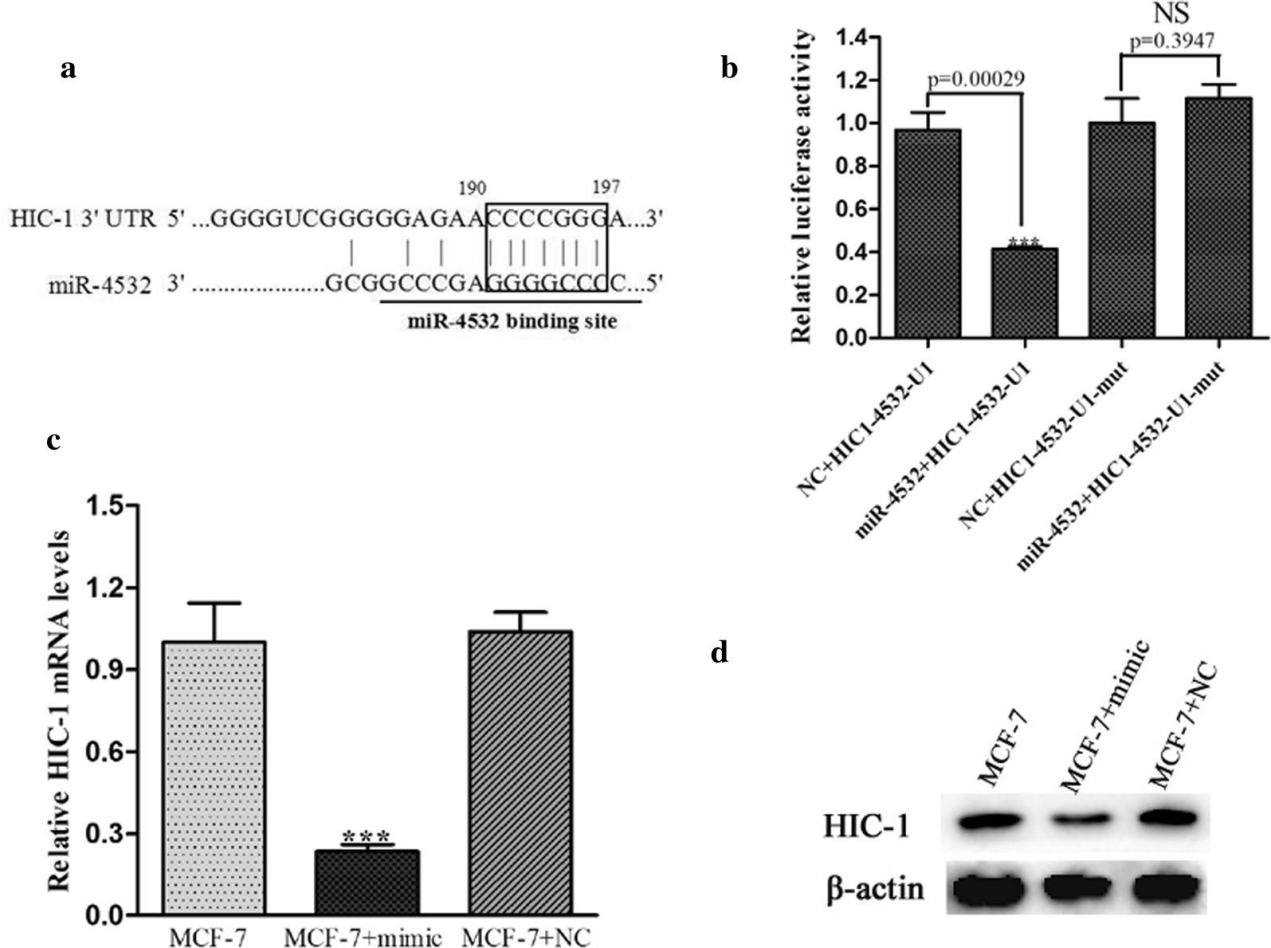
*miR*-*4532* suppressed the expression of the *HIC*-*1* gene. **a** Sequence alignment between *miR*-*4532* and the 3′-UTR of *HIC*-*1*. **b** Effects of *miR*-*4532* on *HIC*-*1* were assessed with the luciferase reporter system. The *miR*-*4532* mimic, together with the luciferase reporter vector or control vector, was transfected into MCF-7 cells (NS: not significant, Student’s *t*-test). **c** qPCR was used to measure the expression levels of *HIC*-*1* mRNA in MCF-7 cells and MCF-7 cells transfected with the *miR*-*4532* mimic or negative control (***p < 0.01, Student’s *t*-test). **d** Western blot analysis of HIC-1 protein expression in MCF-7 cells and MCF-7 cells transfected with the *miR*-*4532* mimic or negative control

To explore the adriamycin-resistance mechanism regulated by *miR*-*4532* in breast cancer cells, target gene prediction was performed using TargetScan and miRanda, and *HIC*-*1* was found to be a potential target gene of *miR*-*4532* (Fig. 4a). To further confirm that *HIC*-*1* was a target of *miR*-*4532*, *miR*-*4532* mimic or negative control miRNA and the luciferase reporter plasmid with the 3′-UTR of *HIC*-*1* were transfected into 293-T cells, in which the target site (GGGGAGAACCCCGGG) is located at the 190th base of the *HIC*-*1* gene 3′-UTR region (U1). As shown in Fig. 4b, the luciferase activity of the 3′-UTR of *HIC*-*1* was significantly suppressed by the *miR*-*4532* mimic, whereas the mutant *HIC*-*1* 3′-UTR (CGGGACAAGGGGCCC) remained unchanged in cells transfected with *miR*-*4532* mimic. These results indicated that *HIC*-*1* was likely to be a target of *miR*-*4532*.

Finally, we examined whether *miR*-*4532* could regulate HIC-1 expression in MCF-7 cells using RT-PCR and western blotting. The results showed that the *miR*-*4532* mimic could downregulate HIC-1 expression in MCF-7 cells (Fig. 4c, d), indicating that there was a consistent and strong inverse correlation between *miR*-*4532* levels and HIC-1.

### HIC-1 inhibited cell migration and invasion in breast cancer cells in vitro

**Fig. 5:**
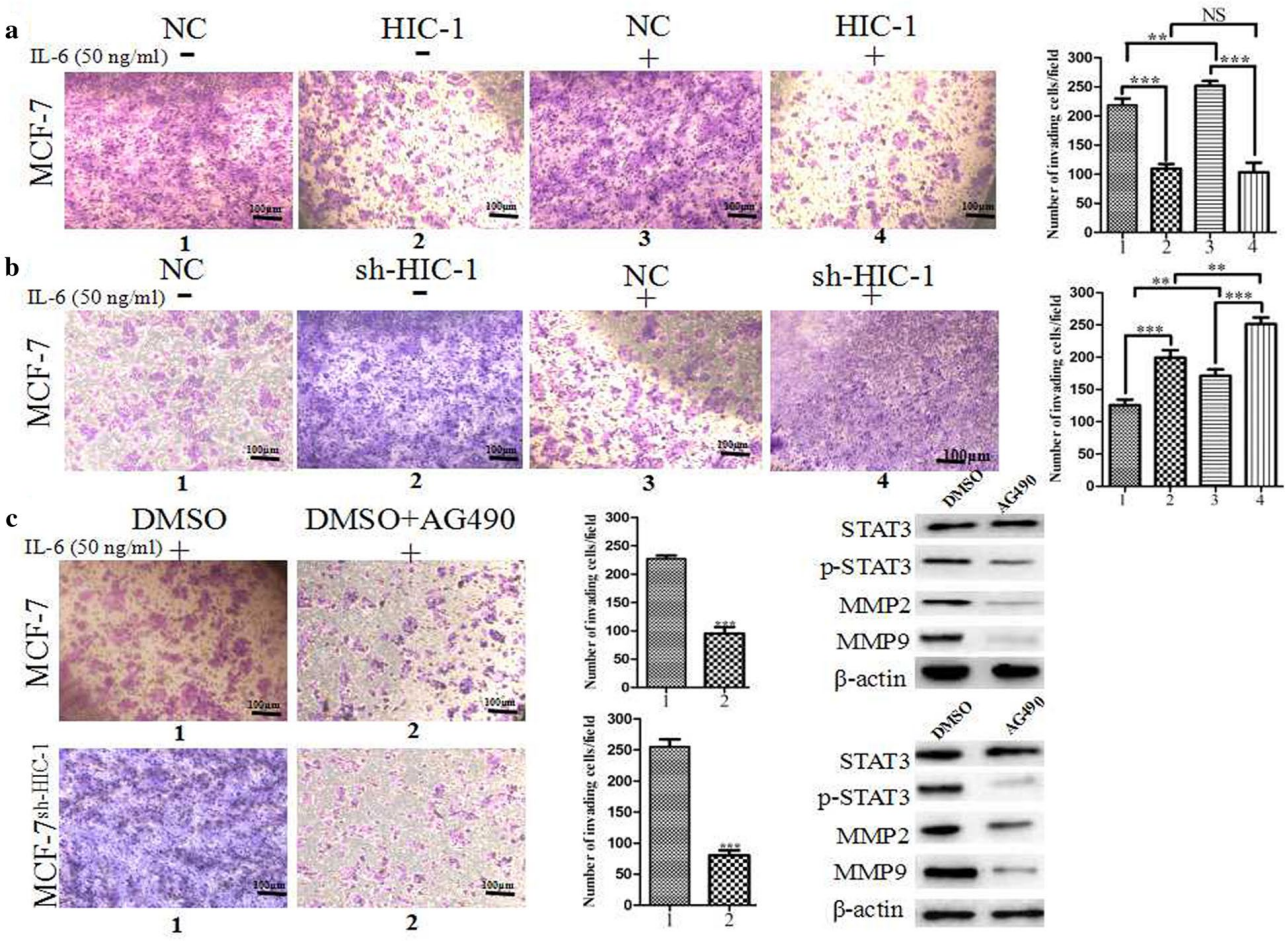
HIC-1 inhibited breast cancer cell invasion and metastasis in vitro. **a** In vitro transwell assays for MCF-7^HIC−1^ and MCF-7^NC^ cells with or without IL-6 stimulation. **b** In vitro transwell assays for MCF-7^sh-HIC−1^ and MCF-7^NC^ cells with or without IL-6 stimulation. **c** In vitro transwell assay for MCF-7 and MCF-7^sh-HIC−1^ cells treated with the STAT3 inhibitor AG490 (100 μM), and western blot analysis of proteins in the STAT3 signaling pathway. The invading cells were observed and analyzed using a microscope with a ×10 objective. Student’s *t*-tests: NS: *p* > 0.05, **p < 0.05, ***p < 0.01, SPSS

Because HIC-1 silencing has been shown to be significantly associated with the clinical features of cancer, including survival, pathological stage, and prognosis, these features maybe closely related to cancer drug resistance, invasion, and metastasis. Hence, an experiment was designed to investigate the effects of HIC-1 on these important events in vitro, as shown in Fig. 5. Overexpression of HIC-1 in MCF-7 cells markedly attenuated cell invasion in transwell assays, despite interleukin (IL)-6-mediated signal transducer and activator of transcription 3 (STAT3) activation. Moreover, knockdown of HIC-1 in MCF-7 cells significantly promoted cell invasion, and this effect was enhanced by IL-6 stimulation due to lack of HIC-1. Treatment with the STAT3 inhibitor AG490 (100 μM) markedly suppressed cell invasion. Interestingly, HIC-1 functioned as a STAT3 inhibitor, similar to AG490, during cell invasion. These results indicated that HIC-1 attenuated cell invasion, even in the presence of IL-6, which activates the STAT3 pathway.

### Low expression of HIC-1 in clinical breast tumors and its effects on patient survival

**Fig. 6:**
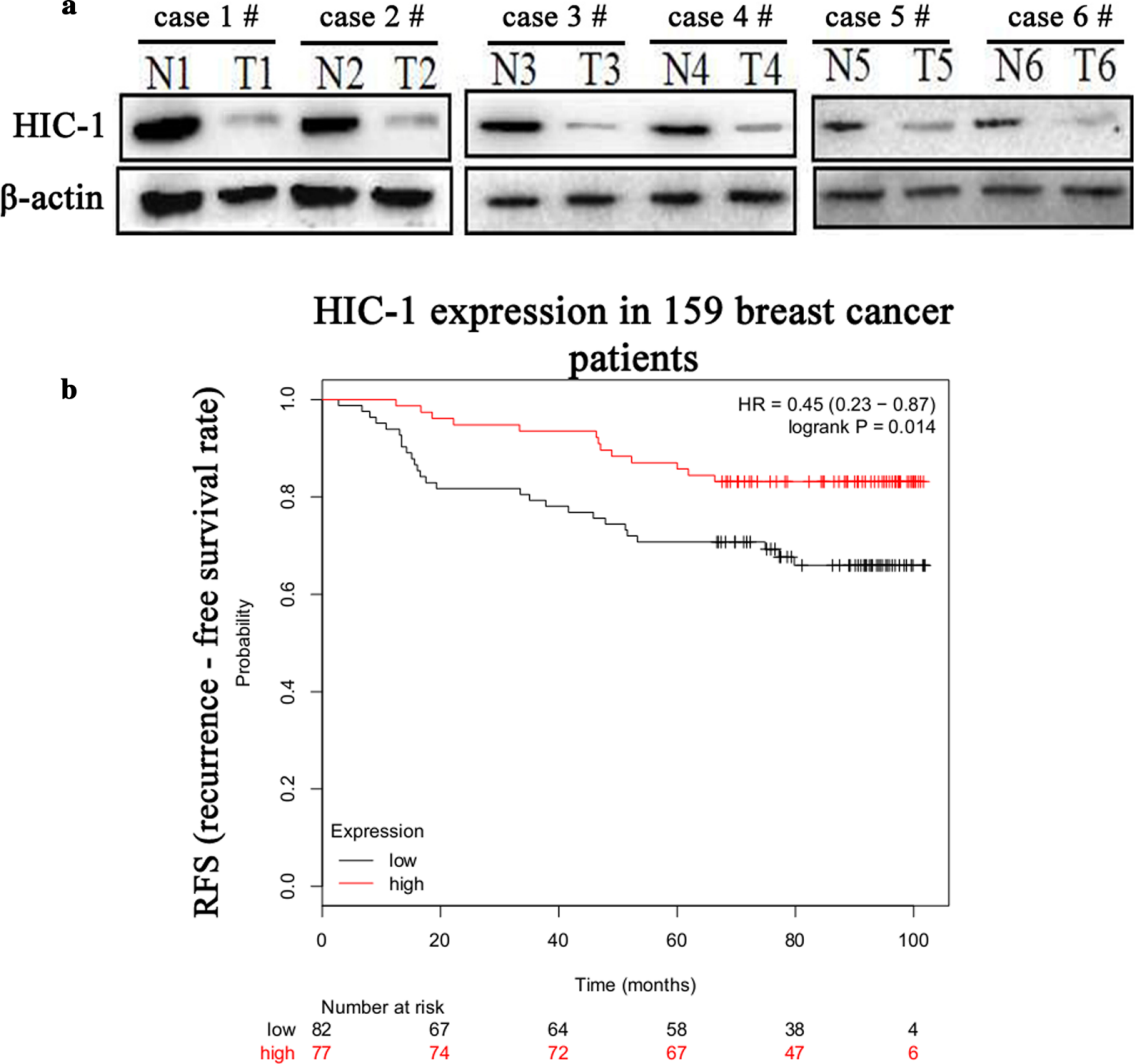
HIC-1 was downregulated in human breast cancer tissue specimens, predicting poor survival and prognosis. **a** Western blot analysis of HIC-1 (90 kDa) in breast cancer tissue specimens. **b** Kaplan–Meier plots of recurrence-free survival (RFS) from data in the dataset GSE1456, stratified by HIC-1 expression. The *p* value was calculated using a log-rank test

Previous studies have reported that the expression of HIC-1 is decreased in many human cancers owing to promoter hypermethylation. Therefore, we examined HIC-1 expression in 10 pairs of breast tumors and matched adjacent nontumor tissues. As shown in Fig. 6a, HIC-1 protein was downregulated in all breast cancer tissues. To further determine the relationship between HIC-1 expression and the clinical prognosis of patients with breast cancer, we evaluated the prognostic value of HIC-1 in a public clinical microarray database of breast cancer cases collected between and 2005 from 2017. Without adjusting for ER, PR, HER2, lymph node metastasis, and TP53 status in patients with breast cancer, the HIC-1 expression level was closely related to recurrence-free survival rate (RFS) in the dataset GSE1456. As shown in Fig. 6b, Kaplan–Meier analysis of 159 patients with breast cancer in Sweden demonstrated that high HIC-1 expression was related to a high RFS rate compared with low HIC-1 expression (*p *= 0.0141).

## Discussion

Breast cancer is a highly heterogeneous disease with a complex etiology involving genetic and environmental factors. Despite the rapid development of molecular diagnosis and chemotherapy for breast cancer, MDR remains an obstacle to effective treatment [34]. Previous functional studies have indicated that miRNAs can regulate almost every cellular process, including MDR; however, the roles of miRNAs in MDR remain largely unknown [35, 36].

In this paper, *miR*-*4532* was found to be expressed in breast cancer cells, and high expression of this miRNA was closely related to drug resistance in breast cancer. Moreover, our analysis showed that *miR*-*4532* regulated *HIC*-*1*, a transcriptional repressor involved in the regulation of cell survival, growth control, and DNA damage response. Finally, we also showed that *miR*-*4532* was upregulated in human breast cancers. However, the biological relationship between *miR*-*4532* and its specific target *HIC*-*1* in adriamycin resistance in MCF-7/ADR cells remains unknown; further studies are needed to elucidate this mechanism.

*HIC*-*1* is a tumor-suppressor gene that is frequently epigenetically silenced or deleted in many human cancers [37]. Growing evidence has suggested that the protein expression level of HIC-1 is correlated with prognosis in patients with cancer [38, 39]. For example, HIC-1 mRNA and protein levels were reported to be low or absent in pancreatic ductal adenocarcinoma (PDAC) tissues, and its expression gradually decreased during the progression of PDAC; negative HIC-1 expression predicted advanced pathological stage and poor patient survival [25]. Moreover, HIC-1 expression was found to be silenced in triple-negative breast cancer [24]. Although studies have identified HIC-1 as having frequent changes in hypermethylation or loss of heterozygosity in many human cancers [40, 41], the molecular mechanisms through which HIC-1 inhibits cancer progression remain poorly understood. HIC-1 is a multifunctional, sequence-specific transcriptional repressor that interacts with several major repression and chromatin remodeling complexes [42, 43]. To date, many studies have showed that the IL-6/Janus kinase/STAT3 signaling pathways are involved in drug resistance, angiogenesis, migration, and other processes in cancers [44, 45]. IL-6-induced transcriptional factor or cytokines, such as c-Myc and vascular endothelial growth factor, initiate and promote cell growth by triggering proliferation, and MMP2 and MMP9 proteins induce cell migration, which is closely related to metastasis and invasiveness in human cancer [46, 47]. Consistent with this, we found, for the first time, that HIC-1 negatively regulated the expression of STAT3, which decreased transcriptional activation of STAT3, and this may explain why HIC-1 acted as an independent prognostic predictor of poor survival.

In summary, in this study, we found that *miR*-*4532* targeted HIC-1 to modulate drug resistance and cell migration. HIC-1 was also found to be differentially expressed in breast cancer tissues and to be correlated with prognosis. Our findings are the first to demonstrate the interactions between *miR*-*4532* and its target *HIC*-*1* in the context of chemotherapeutic drug resistance in breast cancer. Accordingly, our results provide new mechanistic insights into the functions of miRNAs as potential therapeutic targets for overcoming MDR in breast cancer.

## Conclusions

The present study firstly indicates the inverse correlation of *miR*-*4532* and HIC-1 in breast cancer cells. Our study show that *miR*-*4532* regulates HIC-1 and the HIC-1-mediated multidrug resistance formation by directly overexpression of *miR*-*4532.* These results suggest that *miR*-*4532* is a regulator of HIC-1 mediated proliferation promoting and anti-apoptosis in breast cancer cells. HIC-1 plays a important role in the physiological regulation of multidrug resistance through the IL-6/STAT3 signal pathway, and has been implicated in the other various tumor. Our data suggests that HIC-1 regulated by *miR*-*4532* is one of the important factors that promotes drug resistance in breast tumor, which also has been demonstrated in the clinical samples and the database of public microarray datasets. In brief, the novel information regarding the link between *miR*-*4532* and HIC-1 in breast cancer cells would be beneficial for the better understanding of drug resistance formation of breast tumor, which provides a novel strategy for clinical application in the future.

## Additional files

**Additional file 1.** Additional table.

**Additional file 2.** miRNA primers used for RT-PCR.

**Additional file 3.** Additional figure.

## Abbreviations

HIC-1: hypermethylated in cancer-1
MDR: multidrug resistance
FCM: flow cytometry
IL-6/STAT3: interleukin-6/signal transducer and activator of transcription 3
qRT-PCR: quantitative real-time polymerase chain reaction
