# Supplementary material for: Downregulation of hypermethylated in cancer-1 by miR-4532 promotes adriamycin resistance in breast cancer cells
Source: Cancer Cell Int. 2018 Sep 4;18:127. doi: 10.1186/s12935-018-0616-x (PMC6123967; doi:10.1186/s12935-018-0616-x)
Supplement: Supplementary file 2 — Additional file 2. miRNA primers used for RT-PCR. [file 12935_2018_616_MOESM2_ESM.docx]

Table S2 miRNA primers used for RT-PCR

miRNA Name Sequences

| hsa-miR-30c-5p | FORWARD | GCGCGGTGTAAACATCCTACACT |
| --- | --- | --- |
| hsa-miR-30c-5p | REVERSE | ATCCAGTGCAGGGTCCGAGG |
| hsa-miR-30c-5p | RT primer | GTCGTATCCAGTGCAGGGTCCGAGGTATTCGCACTGGATACGACGCTGAG |

| hsa-miR-30b-5p | FORWARD | CGGACGGTGTAAACATCCTACAC |
| --- | --- | --- |
| hsa-miR-30b-5p | REVERSE | ATCCAGTGCAGGGTCCGAGG |
| hsa-miR-30b-5p | RT primer | GTCGTATCCAGTGCAGGGTCCGAGGTATTCGCACTGGATACGACAGCTGA |

| hsa-miR-4532 | FORWARD | ACGCGCCCCGGGGAGC |
| --- | --- | --- |
| hsa-miR-4532 | REVERSE | ATCCAGTGCAGGGTCCGAGG |
| hsa-miR-4532 | RT primer | GTCGTATCCAGTGCAGGGTCCGAGGTATTCGCACTGGATACGACCGCCGG |

| hsa-miR-4485-3p | FORWARD | ACGCGTAACGGCCGCGGTA |
| --- | --- | --- |
| hsa-miR-4485-3p | REVERSE | ATCCAGTGCAGGGTCCGAGG |
| hsa-miR-4485-3p | RT primer | GTCGTATCCAGTGCAGGGTCCGAGGTATTCGCACTGGATACGACTTAGGG |

| hsa-miR-6087 | FORWARD | CGCGTGAGGCGGGGGG |
| --- | --- | --- |
| hsa-miR-6087 | REVERSE | ATCCAGTGCAGGGTCCGAGG |
| hsa-miR-6087 | RT primer | GTCGTATCCAGTGCAGGGTCCGAGGTATTCGCACTGGATACGACGCTCGC |
