# Supplementary figures and images for: Downregulation of hypermethylated in cancer-1 by miR-4532 promotes adriamycin resistance in breast cancer cells
Source: Cancer Cell Int. 2018 Sep 4;18:127. doi: 10.1186/s12935-018-0616-x (PMC6123967; doi:10.1186/s12935-018-0616-x)

**Additional figure**

**1 2 3**


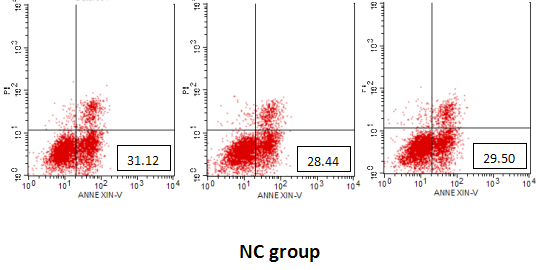


**1 2 3**


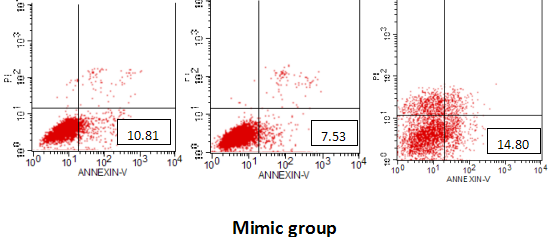

Supplement: Supplementary file 3 — Additional file 3. Additional figure. [file 12935_2018_616_MOESM3_ESM.doc]
